# Short-term effects of air pollution and weather changes on the occurrence of acute aortic dissection in a cold region

**DOI:** 10.3389/fpubh.2023.1172532

**Published:** 2023-08-02

**Authors:** Haiyu Zhang, Leilei Yin, Yingtao Zhang, Zhaowen Qiu, Sizheng Peng, Zhonghua Wang, Bo Sun, Jianrui Ding, Jing Liu, Kai Du, Mingxin Wang, Yanming Sun, Jing Chen, Hongyan Zhao, Tao Song, Yuhui Sun

**Affiliations:** ^1^Department of Cardiology, First Affiliated Hospital of Harbin Medical University, Harbin, Heilongjiang, China; ^2^Department of Emergency, The First Affiliated Hospital of Harbin Medical University, Harbin, China; ^3^School of Computer Science and Technology, Harbin Institute of Technology, Harbin, China; ^4^School of Information and Computer Engineering, Northeast Forestry University, Harbin, Heilongjiang, China; ^5^School of Computer Science and Technology, Harbin Institute of Technology, Weihai, Shandong, China; ^6^Department of Cardiology, Harbin Second Hospital, Harbin, China; ^7^Department of Medical Record, The Second Affiliated Hospital of Harbin Medical University, Harbin, China; ^8^Department of Obstetrics and Gynecology, First Affiliated Hospital of Harbin Medical University, Harbin, Heilongjiang, China

**Keywords:** weather, environment, temperature, pollution, acute aortic dissection, COVID-19

## Abstract

**Background:**

Air pollution and severe weather conditions can adversely affect cardiovascular disease emergencies. Nevertheless, it remains unclear whether air pollutants and low ambient temperature can trigger the occurrence of acute aortic dissection (AAD) in cold regions.

**Methods:**

We applied a retrospective analysis to assess the short-term effects of air pollution and ambient temperature on the occurrence of AAD in Harbin, China. A total of 564 AAD patients were enrolled from a major hospital in Harbin between January 1, 2017, and February 5, 2021. Weather condition data and air pollutant concentrations, including fine particulate matter smaller than 10 μm (PM_10_) and 2.5 μm in diameter (PM_2.5_), nitrogen dioxide (NO_2_), sulfur dioxide (SO_2_), carbon monoxide (CO), and ozone (O_3_), were collected every day. Conditional logistic regressions and correlation analysis were applied to analyze the relationship of environmental and atmospheric parameters with AAD occurrence at lags of 0 to 7 days. Specifically, we appraised the air quality index, CO, NO_2_, SO_2_, O3, PM_10_, PM_2.5_, temperature, dew point temperature, atmospheric pressure, and cloud amount.

**Results:**

A total of 1,496 days at risk were assessed, of which 564 patients developed AAD. Specifically, AAD did not occur on 1,043 (69.72%) days, while 1 or more cases occurred on 453 (30.28%) days. Several pollution and weather predictors for AAD were confirmed by multilevel modeling. The air quality index (*p* = 0.0012), cloud amount (*p* = 0.0001), and concentrations of PM_2.5_ (*p* = 0.0004), PM_10_ (*p* = 0.0013), NO_2_ (*p* = 0.0007) and O_3_ (p = 0.0001) predicted AAD as early as 7 days before the incident (lag of 7 days) in the study period. However, only concentrations of the air pollutants NO_2_ (*p* = 0.0468) and O_3_ (*p* = 0.011) predicted the occurrence of AAD after the COVID-19 outbreak. Similar predictive effects were observed for temperature, dew point temperature, and atmospheric pressure (all *p* < 0.05) on all days.

**Conclusion:**

The risk of AAD is closely related to air pollution and weather characteristics in Harbin. While causation was not determined, the impact of air pollutants on the risk of AAD was reduced after the COVID-19 outbreak.

## Introduction

Acute aortic dissection (AAD) is the most common medical emergency in aortic diseases and is caused by tearing of the lining of the aorta. If left untreated, the mortality rate in the first 24 h after the onset of aortic dissection increases by 1 to 2% per hour (1, 2). According to the 2010 Global Burden of Disease (GBD) data, the total global mortality rate for aortic aneurysms and AAD increased by 0.29 per 100,000 from 1990 to 2010. Specifically, the mortality rate increased from 2.49 per 100,000 inhabitants to 2.78 per 100,000 inhabitants (3). Therefore, it is particularly important to ascertain potential risk factors for AAD to improve prevention. The public health relevance of a precipitating factor depends on two perspectives: the relative risk of the individual and the prevalence of the precipitating factor in the population (4). The relative risk factors of individuals for AAD include arterial hypertension, bicuspid aortic valve, coarctation of the aorta, and Turner syndrome (5–8). Although the relative risk of AAD is small for a given individual precipitating environmental factors can affect the entire population and have a major public health influence.

Studies have found that the incidence of AAD is significantly associated with the season and is highest in winter and lowest in summer (9–14). In addition, the incidence of AAD is higher in cold regions than in tropical regions. Such seasonal and regional differences indicate that ambient temperature and meteorological variables may be significant environmental risk factors for AAD (15). In addition, the independent and joint effects of ambient weather characteristics and pollution on subsequent AAD risk remain unclear (16, 17). To the best of our knowledge, no studies have examined ambient temperature and air pollutants as underlying risk factors for AAD. Additionally, few studies have assessed time-lag patterns of the impacts of temperature and air pollutants on the incidence of AAD at the individual level in cold regions. Such research could improve early intervention for AAD. Since the outbreak of the coronavirus disease 2019 (COVID-19) pandemic, the global healthcare system has faced unprecedented challenges. COVID-19 has an important impact on the incidence, management and outcome of many cardiovascular diseases (18), among which AAD is a serious but underestimated problem (19). During the COVID-19 pandemic, it has been reported that the surgical volume of acute type A aortic dissection has decreased by 30% (20). A recent study has reported that the COVID-19 has significantly reduced the number of admissions to AAD throughout the year or part of the time, and the in-hospital mortality rate of AAD has increased compared with the pre-pandemic period (21). However, no studies have assessed the effects of population behavior on the occurrence of AAD after the COVID-19 outbreak in China.

Therefore, drawing on data from a regionwide registry in Northeast China, we aimed to determine the influence of meteorological factors, including ambient temperature and air pollutants, on the risk of AAD occurrence in a cold region. In this region, the heating period lasts for 6 months, and the minimum temperature in winter is even lower to −20°C, these conditions may heighten the risk of AAD in the days following particular pollutant and weather variations. Furthermore, the relevant factors for AAD before and after the COVID-19 outbreak would also be explored in this study.

## Materials and methods

This was a retrospective analysis of anonymized data from the emergency department of the First Affiliated Hospital of Harbin Medical University, the largest heart disease center in Heilongjiang Province, China. Data were collected between January 1, 2017, and February 5, 2021. Specifically, patients with AAD were identified in a retrospective manner by examining hospital admission diagnosis records and surgical and echocardiographic databases. A diagnosis of AAD was verified through computed tomography with arterial contrast, magnetic resonance imaging, or echocardiography. All emergency admissions with a new episode of AAD met the eligibility criteria. The exclusion criteria were as follows: (1) traumatic AAD, (2) iatrogenic aortic disease secondary to previous cardiac surgery or interventional repair, (3) unidentified AAD with no evidence on multidetector computed tomography scans or echocardiography, (4) subacute and chronic aortic dissection, or (5) not living in this cold region. The numbers of daily AAD cases were entered in a database containing data on local weather and pollution acquired from the China Integrated Meteorological Information Service System (CIMISS), provided by the China Meteorological Data Service Center. These meteorological conditions and air pollutants data were sourced from 31 national meteorological observation stations within 200 kilometers around Harbin, including 2 base stations, 10 basic stations, and 19 general stations. All included patients lived in a cold region to minimize climatic variation among study regions. This study was approved by the Ethics Committee of the First Affiliated Hospital of Harbin Medical University, and the procedures were carried out according to the approved guidelines.

Data on the following environmental contaminants were systematically collected several times a day: fine particulate matter [i.e., with a mean aerodynamic diameter < 2.5 μm (PM_2.5_)] and particulate matter with a mean aerodynamic diameter ≥ 2.5 μm and < 10 μm (PM_10_), nitrogen dioxide (NO_2_), sulfur dioxide (SO_2_), carbon monoxide (CO), and ozone (O_3_). All concentrations are expressed as μm/m^3^. Data on these pollutants were collected every 3 h throughout the region and minimum, maximum, average, and change values were recorded. At the same time, we systematically collected the following weather characteristics: temperature (°C), dew point temperature, atmospheric pressure, and cloud amount. Similarly, we collected minimum, maximum, average, and variation values for all of the above weather characteristics (except cloud amount) every 3 h. Our explicit objective was to assess the impacts of pollution and weather characteristics on AAD occurrence on the same day as well as 1, 2, 3, 5, and 7 days later.

Categorical variables are presented as counts and percentages. Continuous variables are presented as the mean ± standard deviation as well as the median and quartiles. Binary logistic regression analyzes were used to determine odds ratios (ORs) and their 95% confidence intervals (CIs). The independent variables in logistic regression models included air quality index, PM2.5, PM10, sulfur dioxide, nitrogen dioxide, ozone, carbon monoxide, temperature, dew point temperature, atmospheric pressure, cloud amount. The effect of COVID-19 outbreak on the relationship between climate state and AAD occurrence was analyzed with interaction and stratified analysis by logistic regression models. We used a 4-knot restricted cubic spline (RCS) to flexibly simulate the correlations of temperature, PM_2.5_, and PM_10_ with AAD. The median of each measure was set as the reference. A *p* value <0.05 was considered statistically significant. Computations were performed with SAS 9.4 (SAS Inc., Cary, N.C., United States) and R 3.5.3 (R Foundation for Statistical Computing, Vienna, Austria).

## Results

**Figure 1:**
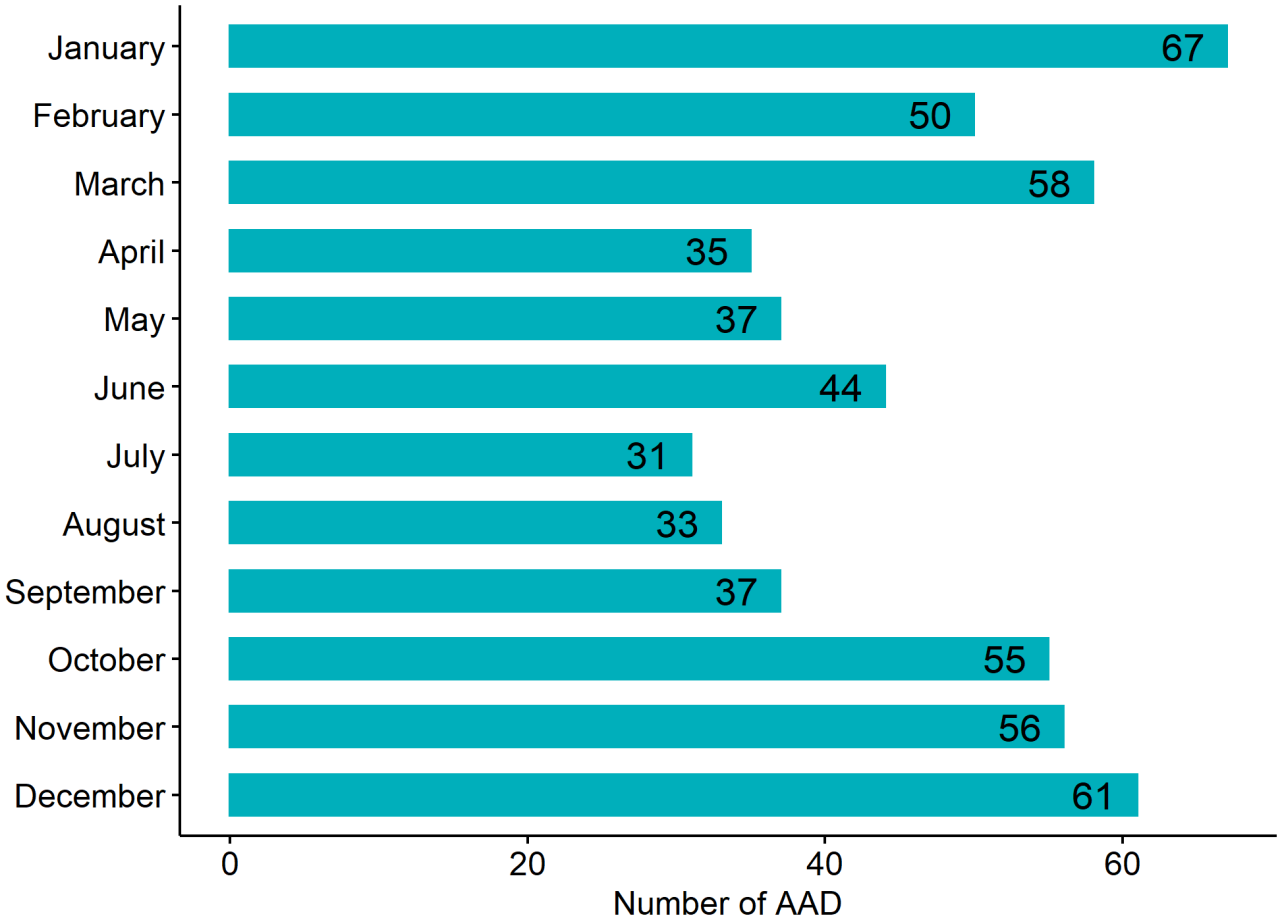
Monthly AAD case numbers from 2017 to 2021 in a cold region.

A total of 1,496 days of risk assessment were included, of which 564 cases (53.37% Stanford type A) of AAD were identified (Table 1). Specifically, there were 1,043 (69.72%) days without an AAD occurrence, 360 (24.06%) days with 1 case, 79 (5.28%) days with 2 cases, and 14 (0.94%) days with three or more cases. Descriptive analysis shows that the quartile variation is large, indicating substantial changes in pollution and weather characteristics. The numbers of AAD cases in each month from 2017 to 2021 in the cold region are shown in Figure 1. As shown in the figure, the AAD case numbers were relatively low in summer and relatively high in winter. In particular, the occurrence of AAD varied greatly between the indoor heating period and nonheating period in the cold region.

**Table 1:** Descriptive analysis.

Feature	*n* (%) or mean ± SD	Median (Q1, Q3)
Daily AAD		
0	1,043 (69.72)	
1	360 (24.06)	
2	79 (5.28)	
3 or more	14 (0.94)	
Air quality index	72.27 ± 57.85	54.13 (37.27, 84.38)
PM2.5, μm/m^3^	47.36 ± 56.41	28.6 (16, 56.85)
PM10, μm/m^3^	71.77 ± 59.03	54.31 (36.27, 86.35)
Sulfur dioxide, μm/m^3^	20.18 ± 15.93	13.38 (8.92, 26.87)
Nitrogen dioxide, μm/m^3^	36.31 ± 16.48	32.78 (24.72, 44.17)
Ozone, μm/m^3^	55.37 ± 24.67	52.94 (36.2, 69.44)
Carbon monoxide, μm/m^3^	0.85 ± 0.42	0.71 (0.57, 0.98)
Temperature, °C	4.81 ± 15.36	6.99 (−10, 18.71)
Dew point temperature, °C	−2.11 ± 15.39	−2.81 (−15.51, 12.14)
Atmospheric pressure, Pa	101332.4 ± 2567.4	10,145 (10066.25, 10227.13)
Cloud amount	4.3 ± 2.97	4.75 (0.5, 6.88)

PM10, particulate matter with a diameter between 2.5 μm and 10 μm; PM2.5, particulate matter with a diameter less than 2.5 μm.

**Figure 2:**
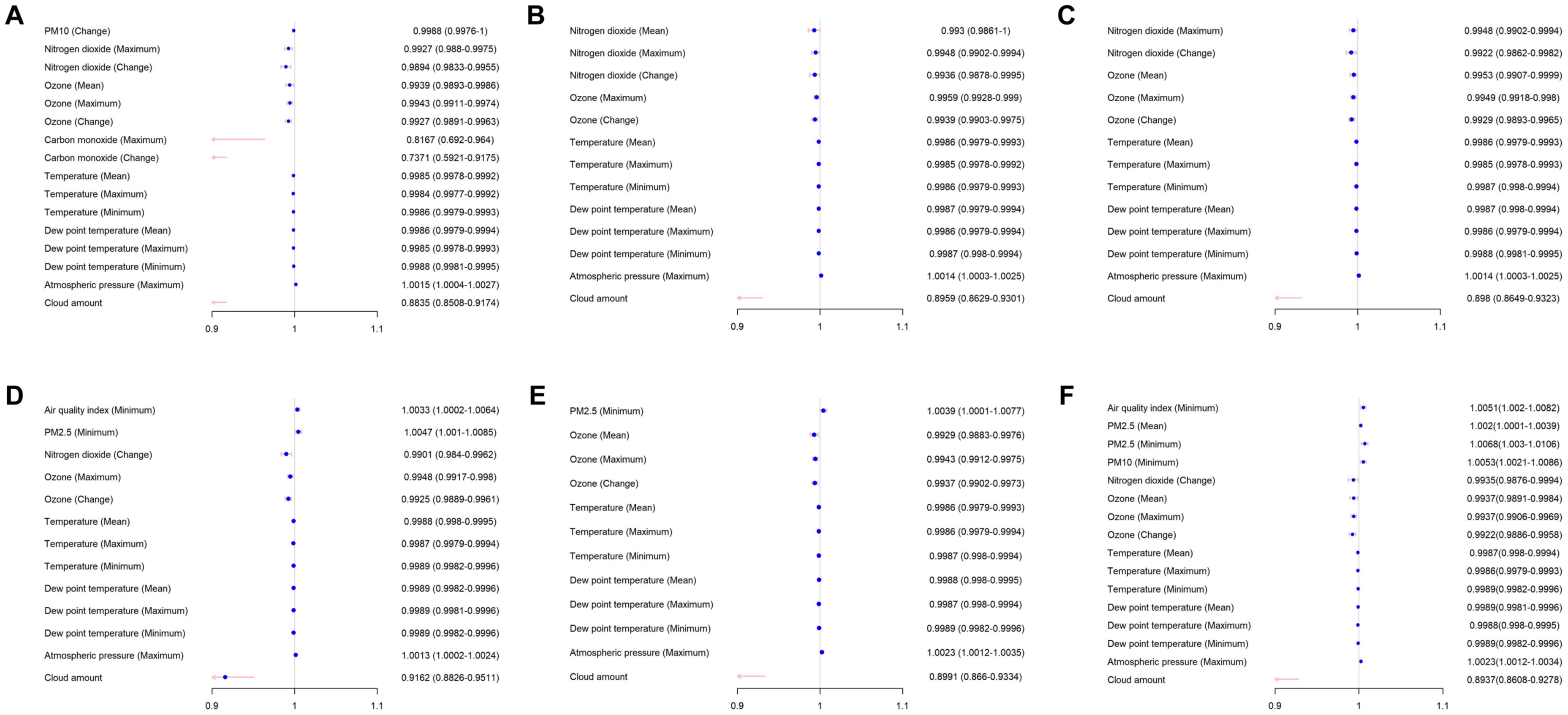
Logistic regression analysis of environmental and weather feature associated with AAD occurrence. The day of onset: **(A)** same day, **(B)** one day before (day – 1), **(C)** two days before (day – 2), **(D)** three days before (day – 3), **(E)** five days before (day – 5), and **(F)** seven days before (day – 7).

Logistic regression models were performed to identify several pollutants and weather characteristics associated with AAD occurrence (Table 2 and Figure 2). These predictors either originated on the same day or the days before AAD occurrence. The air quality index (*p* = 0.0012), cloud amount (*p* = 0.0001), and concentrations of PM2.5 (*p* = 0.0004), PM10 (*p* = 0.0013), NO2 (*p* = 0.0007) and O3 (p = 0.0001) predicted AAD occurrence at lags of 0 to 7 days. Similarly, temperature, dew point temperature, and atmospheric pressure (all *p* < 0.05) significantly predicted AAD occurrence. Notably, temperature, dew point temperature, and O3 were negatively correlated with the risk of AAD occurrence; days with low temperature, low dew point temperature, and low O3 concentration were associated with a higher risk of AAD occurrence at lags of 0 to 7 days.

**Table 2:** Exploratory inferential analysis of data in all days.

Daily environmental or weather feature	Same day	Day-1	Day-2	Day-3	Day-5	Day-7
Air quality index						
Mean	*p* = 0.8925	*p* = 0.8435	*p* = 0.5191	*p* = 0.0688	*p* = 0.0921	*p* = 0.0189^a^
Maximum	*p =* 0.3239	*p* = 0.8789	*p =* 0.8235	*p* = 0.242	*p* = 0.1026	*p* = 0.2304
Minimum	*p =* 0.2898	*p* = 0.9425	*p* = 0.4971	*p* = 0.0367^a^	*p* = 0.1105	*p* = 0.0012^a^
Change	*p* = 0.0967	*p* = 0.8259	*p* = 0.9951	*p* = 0.5615	*p* = 0.1725	*p* = 0.9033
PM2.5						
Mean	*p* = 0.8887	*p* = 0.8932	*p* = 0.4787	*p* = 0.0995	*p* = 0.2538	*p* = 0.0381^a^
Maximum	*p* = 0.3299	*p* = 0.9867	*p* = 0.8585	*p* = 0.4443	*p* = 0.5554	*p* = 0.2976
Minimum	*p* = 0.0617	*p* = 0.8134	*p* = 0.1963	*p* = 0.0139^a^	*p* = 0.0444^a^	*p* = 0.0004^a^
Change	*p* = 0.1232	*p* = 0.9702	*p* = 0.6058	*p* = 0.7854	*p* = 0.8527	*p* = 0.7511
PM10						
Mean	*p* = 0.3287	*p* = 0.7246	*p* = 0.8202	*p* = 0.2579	*p* = 0.25	*p* = 0.1305
Maximum	*p* = 0.1108	*p* = 0.6224	*p* = 0.3865	*p* = 0.8646	*p* = 0.4008	*p* = 0.7129
Minimum	*p* = 0.5981	*p* = 0.8025	*p* = 0.8139	*p* = 0.1049	*p* = 0.2006	*p* = 0.0013^a^
Change	*p* = 0.0478^a^	*p* = 0.6221	*p* = 0.2874	*p* = 0.7744	*p* = 0.555	*p* = 0.5859
Sulfur dioxide						
Mean	*p* = 0.9428	*p* = 0.6144	*p* = 0.9478	*p* = 0.6227	*p* = 0.4074	*p* = 0.2735
Maximum	*p* = 0.6953	*p* = 0.3725	*p* = 0.5336	*p* = 0.8209	*p* = 0.6776	*p* = 0.6484
Minimum	*p* = 0.4058	*p* = 0.7283	*p* = 0.4203	*p* = 0.177	*p* = 0.0923	*p* = 0.0605
Change	*p* = 0.3221	*p* = 0.148	*p* = 0.1921	*p* = 0.3042	*p* = 0.7744	*p* = 0.741
Nitrogen dioxide						
Mean	*p* = 0.0558	*p* = 0.0499^a^	*p* = 0.1295	*p* = 0.6268	*p* = 0.8721	*p* = 0.8718
Maximum	*p* = 0.0026^a^	*p* = 0.028^a^	*p* = 0.0277^a^	*p* = 0.0424^a^	*p* = 0.5564	*p* = 0.4074
Minimum	*p* = 0.5109	*p* = 0.2869	*p* = 0.7302	*p* = 0.3063	*p* = 0.3805	*p* = 0.0705
Change	*p* = 0.0007^a^	*p* = 0.0346^a^	*p* = 0.0104^a^	*p* = 0.0014^a^	*p* = 0.1981	*p* = 0.0301^a^
Ozone						
Mean	*p* = 0.0108^a^	*p* = 0.0762	*p* = 0.0468^a^	*p* = 0.0525	*p* = 0.0033^a^	*p* = 0.0085^a^
Maximum	*p* = 0.0004^a^	*p* = 0.0093^a^	*p* = 0.0014^a^	*p* = 0.0012^a^	*p* = 0.0004^a^	*p* = 0.0001^a^
Minimum	*p* = 0.7859	*p* = 0.4561	*p* = 0.7115	*p* = 0.496	*p* = 0.2258	*p* = 0.6297
Change	*p* = 0.0001^a^	*p* = 0.0008^a^	*p* = 0.0001^a^	*p* = 0.0001^a^	*p* = 0.0006^a^	*p* = 0.0001^a^
Carbon monoxide						
Mean	*p* = 0.0915	*p* = 0.1009	*p* = 0.2042	*p* = 0.6476	*p* = 0.5723	*p* = 0.783
Maximum	*p* = 0.0167^a^	*p* = 0.1501	*p* = 0.2001	*p* = 0.4717	*p* = 0.7257	*p* = 0.8035
Minimum	*p* = 0.5142	*p* = 0.1055	*p* = 0.2551	*p* = 0.7971	*p* = 0.6507	*p* = 0.3509
Change	*p* = 0.0063^a^	*p* = 0.2784	*p* = 0.2698	*p* = 0.4276	*p* = 0.8134	*p* = 0.9105
Temperature						
Mean	*p* = 0.0001^a^	*p* = 0.0001^a^	*p* = 0.0001^a^	*p* = 0.0008^a^	*p* = 0.0002^a^	*p* = 0.0004^a^
Maximum	*p* = 0.0001^a^	*p* = 0.0001^a^	*p* = 0.0001^a^	*p* = 0.0003^a^	*p* = 0.0002^a^	*p* = 0.0002^a^
Minimum	*p* = 0.0001^a^	*p* = 0.0001^a^	*p* = 0.0003^a^	*p* = 0.0015^a^	*p* = 0.0004^a^	*p* = 0.0013^a^
Change	*p* = 0.2743	*p* = 0.8657	*p* = 0.4964	*p* = 0.148	*p* = 0.9999	*p* = 0.069
Dew point temperature						
Mean	*p* = 0.0003^a^	*p* = 0.0004^a^	*p* = 0.0005^a^	*p* = 0.0031^a^	*p* = 0.0008^a^	*p* = 0.002^a^
Maximum	*p* = 0.0001^a^	*p* = 0.0004^a^	*p* = 0.0004^a^	*p* = 0.003^a^	*p* = 0.0006^a^	*p* = 0.0013^a^
Minimum	*p* = 0.0005^a^	*p* = 0.0002^a^	*p* = 0.0006^a^	*p* = 0.0025^a^	*p* = 0.0015^a^	*p* = 0.0024^a^
Change	*p* = 0.5786	*p* = 0.0511	*p* = 0.6738	*p* = 0.2082	*p* = 0.9309	*p* = 0.8599
Atmospheric pressure						
Mean	*p* = 0.5499	*p* = 0.4324	*p* = 0.2076	*p* = 0.5325	*p* = 0.1651	*p* = 0.2417
Maximum	*p* = 0.0064^a^	*p* = 0.0158^a^	*p* = 0.0158^a^	*p* = 0.0205^a^	*p* = 0.0001^a^	*p* = 0.0001^a^
Minimum	*p* = 0.4022	*p* = 0.812	*p* = 0.9066	*p* = 0.3619	*p* = 0.9191	*p* = 0.4844
Change	*p* = 0.2343	*p* = 0.5735	*p* = 0.6591	*p* = 0.2263	*p* = 0.6511	*p* = 0.2234
Cloud amount	*p* = 0.0001^a^	*p* = 0.0001^a^	*p* = 0.0001^a^	*p* = 0.0001^a^	*p* = 0.0001^a^	*p* = 0.0001^a^

a Relative risk values are statistically significant.

**Figure 3:**
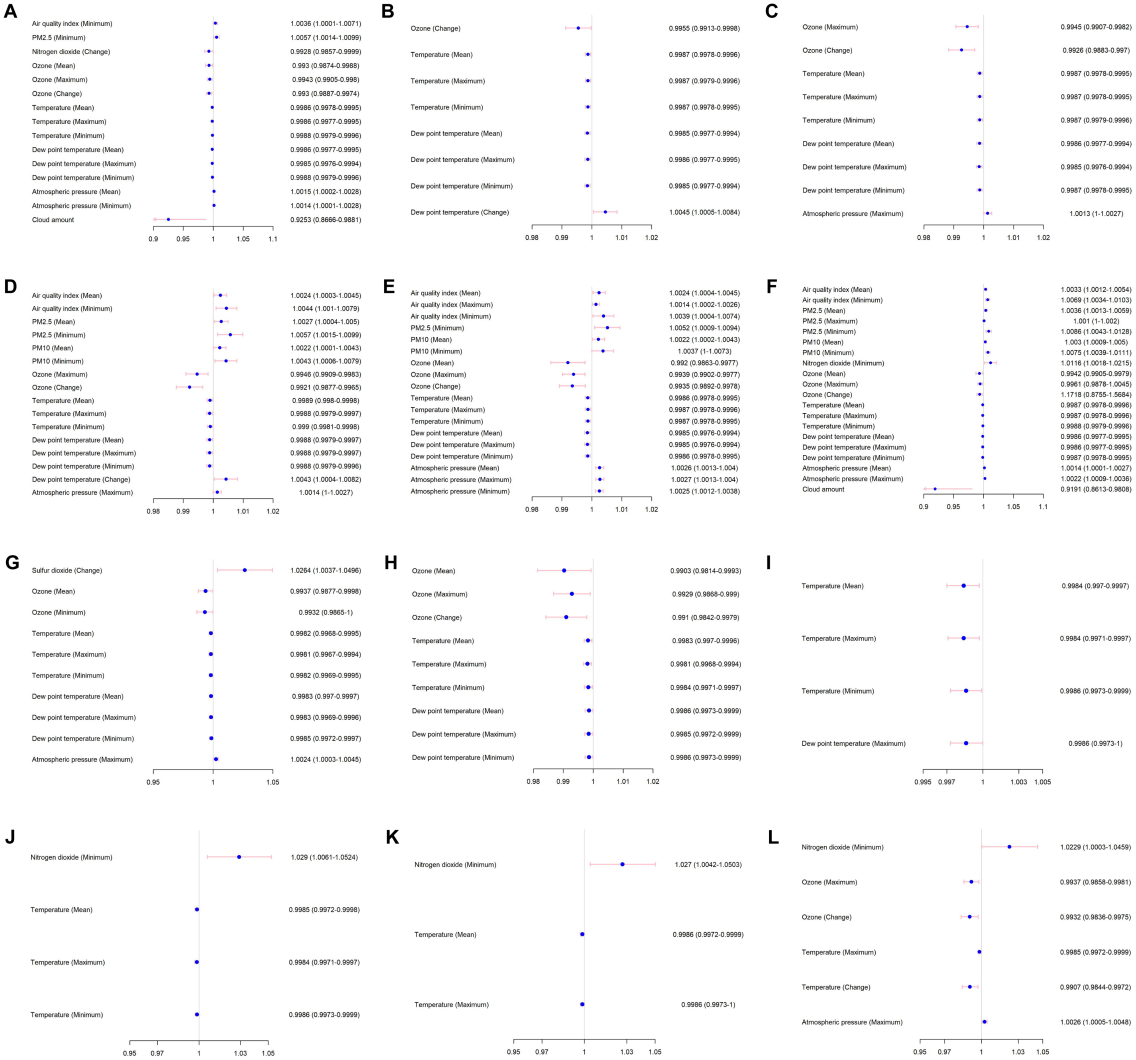
Stratified analysis for COVID-19 outbreak of environmental and weather feature associated with AAD occurrence. The day of onset: **(A)** same day, before February 1, 2020, **(B)** one day before (day – 1), before the COVID-19 outbreak, **(C)** two days before (day – 2), before the COVID-19 outbreak, **(D)** three days before (day – 3), before the COVID-19 outbreak, **(E)** five days before (day – 5), before the COVID-19 outbreak, and **(F)** seven days before (day – 7), before the COVID-19 outbreak. **(G)** same day, after the COVID-19 outbreak, **(H)** one day before (day – 1), after the COVID-19 outbreak, **(I)** two days before (day – 2), after the COVID-19 outbreak, **(J)** three days before (day – 3), after the COVID-19 outbreak, **(K)** five days before (day – 5), after the COVID-19 outbreak, and **(L)** seven days before (day – 7), after the COVID-19 outbreak.

The interactions analyzes showed that the relationship between climate state and AAD occurrence was affected by COVID-19 outbreak (Supplementary Table S1). Subgroup analyzes were then conducted based on whether COVID-19 outbreak (Tables 3, 4 and Figure 3). In the days before the COVID-19 outbreak, the concentrations of pollutants performed a predictive ability significantly on the same day or the days before AAD occurrence. Interestingly, in the days after the COVID-19 outbreak, only concentrations of NO_2_ (*p* = 0.0468) and O_3_ (*p* = 0.011) predicted AAD occurrence as early as 7 days before the event. Temperature, dew point temperature, and atmospheric pressure also exhibited obvious event correlations.

**Table 3:** Exploratory inferential analysis of data before February 1, 2020.

Daily environmental or weather feature	Same day	Day-1	Day-2	Day-3	Day-5	Day-7
Air quality index						
Mean	*p* = 0.3871	*p* = 0.2371	*p* = 0.1300	*p* = 0.0243^a^	*p* = 0.0221^a^	*p* = 0.0018^a^
Maximum	*p* = 0.9116	*p* = 0.2838	*p* = 0.2344	*p* = 0.1259	*p* = 0.0225^a^	*p* = 0.0738
Minimum	*p* = 0.0422^a^	*p* = 0.2890	*p* = 0.1665	*p* = 0.0121^a^	*p* = 0.0295^a^	*p* = 0.0001^a^
Change	*p* = 0.4783	*p* = 0.3740	*p* = 0.3681	*p* = 0.3971	*p* = 0.0547	*p* = 0.5863
PM2.5						
Mean	*p* = 0.2230	*p* = 0.2657	*p* = 0.1055	*p* = 0.0210^a^	*p* = 0.0630	*p* = 0.0019^a^
Maximum	*p* = 0.8672	*p* = 0.3252	*p* = 0.4780	*p* = 0.1299	*p* = 0.1707	*p* = 0.0425^a^
Minimum	*p* = 0.0085^a^	*p* = 0.2398	*p* = 0.0856	*p* = 0.0081^a^	*p* = 0.0166^a^	*p* = 0.0001^a^
Change	*p* = 0.6064	*p* = 0.4187	*p* = 0.7245	*p* = 0.3047	*p* = 0.3535	*p* = 0.2090
PM10						
Mean	*p* = 0.7343	*p* = 0.3342	*p* = 0.2807	*p* = 0.0382^a^	*p* = 0.0345^a^	*p* = 0.0051^a^
Maximum	*p* = 0.8664	*p* = 0.4805	*p* = 0.6909	*p* = 0.3282	*p* = 0.0625	*p* = 0.0965
Minimum	*p* = 0.0964	*p* = 0.3190	*p* = 0.2215	*p* = 0.0210^a^	*p* = 0.0491^a^	*p* = 0.0000^a^
Change	*p* = 0.4903	*p* = 0.6102	*p* = 0.9279	*p* = 0.6691	*p* = 0.1219	*p* = 0.5037
Sulfur dioxide						
Mean	*p* = 0.6882	*p* = 0.9162	*p* = 0.7300	*p* = 0.4349	*p* = 0.2236	*p* = 0.1321
Maximum	*p* = 0.9211	*p* = 0.8506	*p* = 0.9834	*p* = 0.7851	*p* = 0.3201	*p* = 0.2029
Minimum	*p* = 0.4122	*p* = 0.6913	*p* = 0.4856	*p* = 0.2392	*p* = 0.0862	*p* = 0.0645
Change	*p* = 0.7737	*p* = 0.6326	*p* = 0.7378	*p* = 0.8201	*p* = 0.6009	*p* = 0.3938
Nitrogen dioxide						
Mean	*p* = 0.5384	*p* = 0.3683	*p* = 0.5883	*p* = 0.7738	*p* = 0.5136	*p* = 0.4480
Maximum	*p* = 0.1476	*p* = 0.5006	*p* = 0.2744	*p* = 0.2736	*p* = 0.7731	*p* = 0.5779
Minimum	*p* = 0.8084	*p* = 0.8008	*p* = 0.7624	*p* = 0.2086	*p* = 0.2539	*p* = 0.0203^a^
Change	*p* = 0.0477^a^	*p* = 0.4923	*p* = 0.1156	*p* = 0.0284	*p* = 0.7050	*p* = 0.4163
Ozone						
Mean	*p* = 0.0170^a^	*p* = 0.2772	*p* = 0.0593	*p* = 0.0739	*p* = 0.0061^a^	*p* = 0.0156^a^
Maximum	*p* = 0.0029^a^	*p* = 0.0983	*p* = 0.0038^a^	*p* = 0.0047^a^	*p* = 0.0016^a^	*p* = 0.0023^a^
Minimum	*p* = 0.5572	*p* = 0.7554	*p* = 0.9537	*p* = 0.5631	*p* = 0.1742	*p* = 0.3642
Change	*p* = 0.0017^a^	*p* = 0.0393a	*p* = 0.0009^a^	*p* = 0.0004^a^	*p* = 0.0031^a^	*p* = 0.0022^a^
Carbon monoxide						
Mean	*p* = 0.4962	*p* = 0.5205	*p* = 0.7661	*p* = 0.9147	*p* = 0.9460	*p* = 0.2864
Maximum	*p* = 0.2153	*p* = 0.6352	*p* = 0.7658	*p* = 0.9813	*p* = 0.7344	*p* = 0.2608
Minimum	*p* = 0.8979	*p* = 0.4111	*p* = 0.6309	*p* = 0.9917	*p* = 0.9280	*p* = 0.1316
Change	*p* = 0.1310	*p* = 0.8314	*p* = 0.8802	*p* = 0.9801	*p* = 0.6358	*p* = 0.4735
Temperature						
Mean	*p* = 0.0023^a^	*p* = 0.0039^a^	*p* = 0.0030^a^	*p* = 0.0125^a^	*p* = 0.0022^a^	*p* = 0.0033^a^
Maximum	*p* = 0.0024^a^	*p* = 0.0052^a^	*p* = 0.0030^a^	*p* = 0.0092^a^	*p* = 0.0036^a^	*p* = 0.0039^a^
Minimum	*p* = 0.0043^a^	*p* = 0.0025^a^	*p* = 0.0035^a^	*p* = 0.0163^a^	*p* = 0.0025^a^	*p* = 0.0040^a^
Change	*p* = 0.8134	*p* = 0.1039	*p* = 0.7087	*p* = 0.6507	*p* = 0.2783	*p* = 0.5804
Dew point temperature						
Mean	*p* = 0.0022^a^	*p* = 0.0014^a^	*p* = 0.0014^a^	*p* = 0.0068^a^	*p* = 0.0010^a^	*p* = 0.0022^a^
Maximum	*p* = 0.0013^a^	*p* = 0.0020^a^	*p* = 0.0015^a^	*p* = 0.0104^a^	*p* = 0.0009^a^	*p* = 0.0027^a^
Minimum	*p* = 0.0042^a^	*p* = 0.0008^a^	*p* = 0.0020^a^	*p* = 0.0046^a^	*p* = 0.0017^a^	*p* = 0.0019^a^
Change	*p* = 0.5386	*p* = 0.0254^a^	*p* = 0.5329	*p* = 0.0322^a^	*p* = 0.8247	*p* = 0.1293
Atmospheric pressure						
Mean	*p* = 0.0273^a^	*p* = 0.1347	*p* = 0.0508	*p* = 0.0541	*p* = 0.0001^a^	*p* = 0.0323^a^
Maximum	*p* = 0.0579	*p* = 0.0930	*p* = 0.0485^a^	*p* = 0.0446^a^	*p* = 0.0001^a^	*p* = 0.0011^a^
Minimum	*p* = 0.0327^a^	*p* = 0.3119	*p* = 0.0820	*p* = 0.0816	*p* = 0.0002^a^	*p* = 0.5269
Change	*p* = 0.4914	*p* = 0.7326	*p* = 0.6681	*p* = 0.6685	*p* = 0.6658	*p* = 0.3319
Cloud amount	*p* = 0.0205^a^	*p* = 0.1071	*p* = 0.0754	*p* = 0.5540	*p* = 0.0617	*p* = 0.0109^a^

a Relative risk values are statistically significant.

**Table 4:** Exploratory inferential analysis of data after February 2, 2020.

Daily environmental or weather feature	Same day	Day-1	Day-2	Day-3	Day-5	Day-7
Air quality index						
Mean	*p* = 0.6816	*p* = 0.7388	*p* = 0.9973	*p* = 0.2523	*p* = 0.4573	*p* = 0.5876
Maximum	*p* = 0.4561	*p* = 0.9297	*p* = 0.8489	*p* = 0.1971	*p* = 0.4279	*p* = 0.4645
Minimum	*p* = 0.7647	*p* = 0.3294	*p* = 0.9438	*p* = 0.3228	*p* = 0.6140	*p* = 0.5256
Change	*p* = 0.4192	*p* = 0.7529	*p* = 0.7866	*p* = 0.2333	*p* = 0.4360	*p* = 0.5187
PM2.5						
Mean	*p* = 0.4948	*p* = 0.5601	*p* = 0.7524	*p* = 0.7332	*p* = 0.9521	*p* = 0.9469
Maximum	*p* = 0.2723	*p* = 0.5355	*p* = 0.5535	*p* = 0.8936	*p* = 0.8056	*p* = 0.6700
Minimum	*p* = 1.0000	*p* = 0.4116	*p* = 0.6414	*p* = 0.2237	*p* = 0.4888	*p* = 0.3619
Change	*p* = 0.2136	*p* = 0.5765	*p* = 0.4598	*p* = 0.7093	*p* = 0.6886	*p* = 0.5261
PM10						
Mean	*p* = 0.3812	*p* = 0.4581	*p* = 0.4764	*p* = 0.7563	*p* = 0.8148	*p* = 0.7264
Maximum	*p* = 0.1754	*p* = 0.5238	*p* = 0.3669	*p* = 0.7991	*p* = 0.9603	*p* = 0.4346
Minimum	*p* = 0.6775	*p* = 0.3418	*p* = 0.7844	*p* = 0.4675	*p* = 0.5559	*p* = 0.5339
Change	*p* = 0.1358	*p* = 0.6211	*p* = 0.3205	*p* = 0.9230	*p* = 0.8201	*p* = 0.2761
Sulfur dioxide						
Mean	*p* = 0.1348	*p* = 0.2971	*p* = 0.2691	*p* = 0.1166	*p* = 0.1866	*p* = 0.2115
Maximum	*p* = 0.0515	*p* = 0.3060	*p* = 0.2128	*p* = 0.0833	*p* = 0.1061	*p* = 0.4430
Minimum	*p* = 0.3165	*p* = 0.5027	*p* = 0.2252	*p* = 0.1301	*p* = 0.2947	*p* = 0.2184
Change	*p* = 0.0224^a^	*p* = 0.2666	*p* = 0.2742	*p* = 0.1027	*p* = 0.0786	*p* = 0.7209
Nitrogen dioxide						
Mean	*p* = 0.9884	*p* = 0.7163	*p* = 0.5191	*p* = 0.1881	*p* = 0.0457	*p* = 0.3271
Maximum	*p* = 0.4144	*p* = 0.5886	*p* = 0.8808	*p* = 0.6542	*p* = 0.3150	*p* = 0.9439
Minimum	*p* = 0.4506	*p* = 0.4775	*p* = 0.2072	*p* = 0.0129^a^	*p* = 0.0200^a^	*p* = 0.0468^a^
Change	*p* = 0.1768	*p* = 0.3156	*p* = 0.6421	*p* = 0.4581	*p* = 0.9820	*p* = 0.2737
Ozone						
Mean	*p* = 0.1218	*p* = 0.0339^a^	*p* = 0.2131	*p* = 0.1950	*p* = 0.0941	*p* = 0.1134
Maximum	*p* = 0.0426^a^	*p* = 0.0228^a^	*p* = 0.1523	*p* = 0.1048	*p* = 0.0957	*p* = 0.0110^a^
Minimum	*p* = 0.5640	*p* = 0.9785	*p* = 0.8785	*p* = 0.6105	*p* = 0.2959	*p* = 0.7209
Change	*p* = 0.0484^a^	*p* = 0.0111^a^	*p* = 0.1267	*p* = 0.1205	*p* = 0.1877	*p* = 0.0077^a^
Carbon monoxide						
Mean	*p* = 0.8985	*p* = 0.9421	*p* = 0.8251	*p* = 0.1632	*p* = 0.2547	*p* = 0.3612
Maximum	*p* = 0.5933	*p* = 0.8820	*p* = 0.9045	*p* = 0.4392	*p* = 0.3408	*p* = 0.5302
Minimum	*p* = 0.2679	*p* = 0.9509	*p* = 0.6497	*p* = 0.0803	*p* = 0.1675	*p* = 0.2733
Change	*p* = 0.2969	*p* = 0.8386	*p* = 0.9851	*p* = 0.7559	*p* = 0.5047	*p* = 0.7111
Temperature						
Mean	*p* = 0.0065^a^	*p* = 0.0119^a^	*p* = 0.0159^a^	*p* = 0.0284^a^	*p* = 0.0334^a^	*p* = 0.0670
Maximum	*p* = 0.0049^a^	*p* = 0.0054^a^	*p* = 0.0191^a^	*p* = 0.0184^a^	*p* = 0.0441^a^	*p* = 0.0297^a^
Minimum	*p* = 0.0075^a^	*p* = 0.0162^a^	*p* = 0.0297^a^	*p* = 0.0322^a^	*p* = 0.0602	*p* = 0.1380
Change	*p* = 0.8177	*p* = 0.1874	*p* = 0.6629	*p* = 0.5160	*p* = 0.7522	*p* = 0.0047^a^
Dew point temperature						
Mean	*p* = 0.0144^a^	*p* = 0.0382^a^	*p* = 0.0515	*p* = 0.0933	*p* = 0.1370	*p* = 0.1806
Maximum	*p* = 0.0128^a^	*p* = 0.0308^a^	*p* = 0.0437^a^	*p* = 0.0687	*p* = 0.1335	*p* = 0.1067
Minimum	*p* = 0.0168^a^	*p* = 0.0316^a^	*p* = 0.0532	*p* = 0.1114	*p* = 0.1684	*p* = 0.2403
Change	*p* = 0.7795	*p* = 0.5524	*p* = 0.8386	*p* = 0.5867	*p* = 0.8911	*p* = 0.1127
Atmospheric pressure						
Mean	*p* = 0.6876	*p* = 0.4225	*p* = 0.2769	*p* = 0.6295	*p* = 0.5328	*p* = 0.3559
Maximum	*p* = 0.0277^a^	*p* = 0.0508	*p* = 0.1357	*p* = 0.2108	*p* = 0.0854	*p* = 0.0146^a^
Minimum	*p* = 0.5496	*p* = 0.9100	*p* = 0.8865	*p* = 0.4943	*p* = 0.9150	*p* = 0.8958
Change	*p* = 0.4515	*p* = 0.9788	*p* = 0.9705	*p* = 0.4406	*p* = 0.9872	*p* = 0.9650
Cloud amount	*p* = 0.9608	*p* = 0.3940	*p* = 0.1140	*p* = 0.0062	*p* = 0.3175	*p* = 0.5968

a Relative risk values are statistically significant.

**Figure 4:**
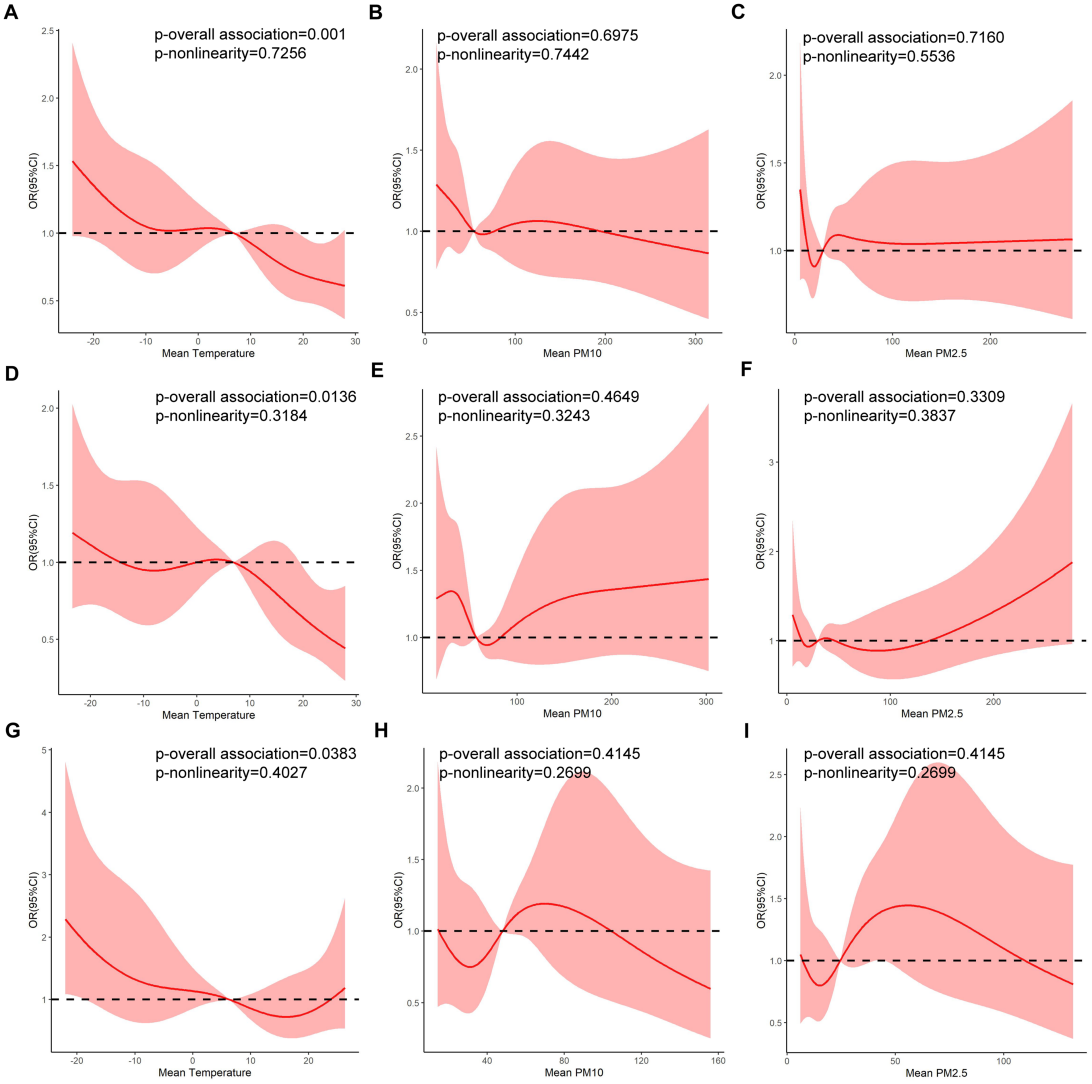
Relationships of mean temperature, mean PM10 concentration and mean PM2.5 concentration with the risk of AAD occurrence for all days, days prior to the COVID-19 outbreak and days after the COVID-19 outbreak. **(A)** Mean temperature: all days. **(B)** PM10 concentration: all days. **(C)** PM2.5 concentration: all days. **(D)** Mean temperature: before the COVID-19 outbreak. **(E)** PM10 concentration: before the COVID-19 outbreak. **(F)** PM2.5 concentration: before the COVID-19 outbreak. **(G)** Mean temperature: after the COVID-19 outbreak. **(H)** PM10 concentration: after the COVID-19 outbreak. **(I)** PM2.5 concentration: after the COVID-19 outbreak (The red solid line is the mean risk estimate, and the red areas are their 95% confidence intervals).

As shown in Figure 4, RCS were used to flexibly model and visualize the correlations of the mean temperature, mean PM_2.5_, and mean PM_10_ with AAD risk. Generally, the correlations were curved rather than linear, indicating the presence of significant quadratic terms and thus the need for visual inspection of quadratic prediction plots. Notably, although there was not a nonlinear relationship between temperature and AAD risk, these variables were significantly related (*p* < 0.05). The median temperature of the mean (6.99°C) was chosen as a reference of RCS in all days. The median temperature of the mean (7.11°C) was chosen as a reference of RCS in days before the COVID-19 outbreak. The median temperature of the mean (6.33°C) was chosen as a reference of RCS in days after the COVID-19 outbreak. The results from RCS showed that as the mean temperature increases, the risk of AAD decreases actually (Figures 4A,D,G).

## Discussion

In this research, a regionwide retrospective study was conducted in Harbin, a large city with the highest latitude and lowest temperatures in Northeast China. Harbin’s climate is characterized by short, cool summers and long, cold winters (22, 23). In addition, the heating period lasts for half a year, during which the outdoor air quality is frequently poor, likely due to fossil fuel combustion for heating, industrial discharge, automobile exhaust, or burning straw in nearby rural areas (24). The impacts of these significant climatic conditions and environmental factors on the occurrence of AAD has not been evaluated previously. Indeed, to the best of our knowledge, this is the first study to examine the effects of daily ambient temperature and air pollutants on the risk of AAD occurrence; additionally, it is the first study to evaluate the influence of the COVID-19 pandemic on the relationship between air pollution and AAD risk in a cold region.

Regarding meteorological indicators, we observed that a drop in temperature was associated with an increased risk of later AAD, with lags of 0 to 7 days. Indicators of this drop in temperature included the daily maximum, minimum, and average temperatures, but did not include the daily temperature change. This association between low temperatures and AAD risk (with lags of 0 to 7 days) was detected even with a long heating period during winter in cold regions. This result aligns with previous findings of associations between low ambient temperature and a high risk of acute cardiovascular incidents, such as acute coronary syndrome, heart failure, hypertension, and stroke (25–29). In addition, the risk of AAD increased with lower ambient temperature to a greater extent than the risks of the cardiovascular events listed above. Regarding the mechanisms by which low temperature can affect AAD risk, there are numerous biological responses to low temperatures, such as increased blood pressure, haemodynamic changes, vasoconstriction in small vessels, behavioral changes, or onset of suboptimal health conditions (13, 15). Nevertheless, there is little evidence of the underlying biological mechanisms of the relationship between low temperatures and an increased risk of AAD.

Dew point temperature had a similar effect on AAD risk as temperature. Dew point temperature is related to the water vapour content (i.e., humidity) in the air; that is, the lower the dew point temperature is, the lower the water vapour content and the lower the relative humidity (30). In this study, we found that a low dew point temperature was associated with a higher risk of later AAD, with lags of 0 to 7 days. Although the underlying mechanism remains unclear, this correlation is also supported by the fact that colder, drier air in winter is associated with a high incidence of AAD in cold regions. On the other hand, it may also indicate that the incidence of AAD in dry inland regions is significantly higher than that in humid coastal regions (31).

Regarding air pollutants, we found that short-term exposure to high concentrations of air pollutants was associated with later AAD risk, with lags of 3 to 7 days. In brief, our results indicate that the risk of AAD is strongly associated with several pollutants and weather characteristics. Our current study was unable to directly investigate the causality of this relationship. However, we found that the following predictors of AAD changed before AAD onset: concentrations of PM_2.5_, PM_10_, NO_2_, O_3_, and CO. These indicators changed drastically as early as 7 days before AAD onset. Increased values of these indicators (other than O_3_) were significantly associated with increased risk of AAD. However, apart from the negative correlation of O_3_ with AAD onset, the risk of AAD was difficult to predict in the short term with pollutant concentrations (i.e., lags varied from 0 to 2 days), especially AQI as a comprehensive indicator.

Fine particulate matter (PM_2.5_) is the most important air pollutant. It is considered the single greatest environmental threat to human health worldwide. Acute cardiovascular events mainly include coronary heart disease, acute myocardial infarction, heart failure, cardiac arrest, hypertension, arrhythmia, and stroke, and according to numerous epidemiological studies, short-term exposure to PM_2.5_ is prominently associated with increased risk of occurrence (1, 4, 32, 33). PM_2.5_ exposure and AAD onset are correlated; moreover, this relationship is also biologically credible. PM_2.5_ exposure increases blood pressure and can thus promote the occurrence of AAD. However, there is a paucity of epidemiological research on the latent risks of AAD associated with PM_2.5_ exposure.

Although the relationship between O_3_ and the occurrence and mortality of cardiovascular diseases is still controversial (34, 35), our results showed that the occurrence of AAD increases with the decreased in O_3_ concentration. Interestingly, results of studies have varied regarding the chronic effects of O_3_ exposure on cardiovascular mortality. There is no clear “safe” threshold for O_3_ exposure in terms of cardiovascular mortality. Long-term exposure to O_3_ is linked to an increased risk of death from cardiovascular disease, ischemic heart disease, and stroke (36, 37). A prior investigation revealed that short-term exposure to O_3_ is linked to higher mortality in those who have previously been hospitalized for acute myocardial infarction (38). In the atmosphere, O_3_ efficiently shields UV rays and safeguards human health; it is typically found approximately 30 kilometers above the ground. However, sufficient light (strong radiation), high temperatures, and low humidity cause photochemical reactions, leading to O_3_ pollution that harms human health and the ecosystem. Although studies have found that O_3_ exposure is associated with cardiovascular mortality (35, 39), the underlying biological mechanism is still unclear. In conclusion, the risk of AAD was inversely related to temperature, dew point temperature, and air pressure, as mentioned previously. In other words, days that were colder, drier, and had lower air pressures, lower O_3_ concentrations, and heavier pollution had a higher risk of AAD.

In addition, after the COVID-19 outbreak, the predictive power of pollutant concentrations on the occurrence of AAD was reduced. The study period lasted 1 year. Except for SO_2_ and O_3_, the pollutants were essentially not associated with the occurrence of AAD, with lags of 0 to 7 days. This may be because people were required to wear masks during the COVID-19 pandemic, which reduced the risk of AAD caused by inhalation of air pollutants. Research has not yet investigated whether wearing masks (N95 masks or even general surgical masks) could prevent cardiovascular emergencies. This may indicate a new avenue for prevention of AAD. Meanwhile, people’s exercise range has been constrained during the COVID-19 preventive and control period, dramatically limiting outdoor activities and vigorous exercise. These characteristics lessen the effect of outdoor air pollution on the occurrence of AAD, and intensive exercise’s effect on blood pressure and heart rate also contributes to the condition’s development.

## Strengths and limitations

To the best of our knowledge, this is the first study to explore the impact of daily ambient temperature and air pollutants on the risk of AAD under low-temperature weather conditions. In this study, we offered novel and convincing evidence in a cold region (Harbin, China), which revealed that short-term exposure to air pollution and low temperatures was linked to an increased risk of AAD. Our findings identify modifiable environmental risk factors for AAD occurrence, which would be useful for AAD prevention, as AAD is a life-threatening cardiovascular emergency.

Due to some limitations, our results should be interpreted with caution. First, this study had a single-center retrospective design, and therefore inevitably included selection bias. Second, there may have been measurement errors due to the collection of meteorological and air pollution data from outdoor monitoring stations. Finally, the interference of indoor temperature and air pollutants was not considered. These factors may be the main causes of cardiovascular emergencies. Most health impacts are due to indoor air pollutants, which can enter a room in various ways from the outdoors (40); therefore, even people who stay indoors most of the time may still be affected by air pollutants.

## Conclusion

The risk of AAD is closely related to air pollution and weather characteristics in Harbin. While causation was not determined, the impact of air pollutants on the risk of AAD was reduced after the COVID-19 outbreak.

## Data availability statement

The raw data supporting the conclusions of this article will be made available by the authors, without undue reservation.

## Ethics statement

Written informed consent was not obtained from the individual(s) for the publication of any potentially identifiable images or data included in this article.

## Author contributions

TS and YuS designed the study, handled data analysis and interpretation, and drafted the manuscript. YZ, ZQ, SP, ZW, BS, JD, JL, KD, MW, YaS, JC, and HoZ participated in the gathering of data, its analysis and interpretation, and its critical examination for key intellectual elements. All authors contributed to the article and approved the submitted version.

## Funding

This work was supported by the Heilongjiang Postdoctoral Research Foundation (LBH-Q20110) and the Scientific Research Fund of the First Affiliated Hospital of Harbin Medical University (HYD2020YQ0004, 2020M20, 2020M08).

## Conflict of interest

The authors declare that the research was conducted in the absence of any commercial or financial relationships that could be construed as a potential conflict of interest.

## Publisher’s note

All claims expressed in this article are solely those of the authors and do not necessarily represent those of their affiliated organizations, or those of the publisher, the editors and the reviewers. Any product that may be evaluated in this article, or claim that may be made by its manufacturer, is not guaranteed or endorsed by the publisher.
