# Supplementary material for: Short-term effects of air pollution and weather changes on the occurrence of acute aortic dissection in a cold region
Source: Front Public Health. 2023 Aug 2;11:1172532. doi: 10.3389/fpubh.2023.1172532 (PMC10433911; doi:10.3389/fpubh.2023.1172532)
Supplement: Supplementary file 1 [file Table_1.DOCX]

Table 1S. Interactions of COVID-19 outbreak on the associations between climate state and AAD.

| Daily environmental or weather feature | Same day | Day +1 | Day +2 | Day +3 | Day +5 | Day +7 |
| --- | --- | --- | --- | --- | --- | --- |
| Air quality index |  |  |  |  |  |  |
| Mean | *p_interaction_ =* 0.4508 | *p_interaction_ =* 0.41 | *p_interaction_ =* 0.5022 | *p_interaction_ =* 0.5022 | *p_interaction_ =* 0.7347 | *p_interaction_ =* 0.3841 |
| Maximum | *p_interaction_ =* 0.4715 | *p_interaction_ =* 0.5907 | *p_interaction_ =* 0.4973 | *p_interaction_ =* 0.4973 | *p_interaction_ =* 0.811 | *p_interaction_ =* 0.9272 |
| Minimum | *p_interaction_ =* 0.2616 | *p_interaction_ =* 0.1862 | *p_interaction_ =* 0.5965 | *p_interaction_ =* 0.5965 | *p_interaction_ =* 0.6446 | *p_interaction_ =* 0.2905 |
| Change | *p_interaction_ =* 0.6754 | *p_interaction_ =* 0.9272 | *p_interaction_ =* 0.5332 | *p_interaction_ =* 0.5332 | *p_interaction_ =* 0.9314 | *p_interaction_ =* 0.7244 |
| PM2.5 |  |  |  |  |  |  |
| Mean | *p_interaction_ =* 0.2203 | *p_interaction_ =* 0.2745 | *p_interaction_ =* 0.2435 | *p_interaction_ =* 0.2435 | *p_interaction_ =* 0.3108 | *p_interaction_ =* 0.0722 |
| Maximum | *p_interaction_ =* 0.2868 | *p_interaction_ =* 0.2991 | *p_interaction_ =* 0.3802 | *p_interaction_ =* 0.3802 | *p_interaction_ =* 0.3346 | *p_interaction_ =* 0.1498 |
| Minimum | *p_interaction_ =* 0.26 | *p_interaction_ =* 0.2162 | *p_interaction_ =* 0.7424 | *p_interaction_ =* 0.7424 | *p_interaction_ =* 0.6904 | *p_interaction_ =* 0.3919 |
| Change | *p_interaction_ =* 0.3502 | *p_interaction_ =* 0.3682 | *p_interaction_ =* 0.4147 | *p_interaction_ =* 0.4147 | *p_interaction_ =* 0.4017 | *p_interaction_ =* 0.2405 |
| PM10 |  |  |  |  |  |  |
| Mean | *p_interaction_ =* 0.3495 | *p_interaction_ =* 0.2742 | *p_interaction_ =* 0.2641 | *p_interaction_ =* 0.2641 | *p_interaction_ =* 0.4422 | *p_interaction_ =* 0.1177 |
| Maximum | *p_interaction_ =* 0.2297 | *p_interaction_ =* 0.3848 | *p_interaction_ =* 0.3243 | *p_interaction_ =* 0.3243 | *p_interaction_ =* 0.4089 | *p_interaction_ =* 0.1753 |
| Minimum | *p_interaction_ =* 0.2984 | *p_interaction_ =* 0.206 | *p_interaction_ =* 0.4595 | *p_interaction_ =* 0.4595 | *p_interaction_ =* 0.8106 | *p_interaction_ =* 0.3082 |
| Change | *p_interaction_ =* 0.2465 | *p_interaction_ =* 0.5111 | *p_interaction_ =* 0.3392 | *p_interaction_ =* 0.3392 | *p_interaction_ =* 0.4106 | *p_interaction_ =* 0.2108 |
| Sulfur dioxide |  |  |  |  |  |  |
| Mean | *p_interaction_ =* 0.1973 | *p_interaction_ =* 0.3072 | *p_interaction_ =* 0.348 | *p_interaction_ =* 0.348 | *p_interaction_ =* 0.3799 | *p_interaction_ =* 0.468 |
| Maximum | *p_interaction_ =* 0.0682 | *p_interaction_ =* 0.3023 | *p_interaction_ =* 0.2385 | *p_interaction_ =* 0.2385 | *p_interaction_ =* 0.2138 | *p_interaction_ =* 0.7237 |
| Minimum | *p_interaction_ =* 0.515 | *p_interaction_ =* 0.6261 | *p_interaction_ =* 0.3724 | *p_interaction_ =* 0.3724 | *p_interaction_ =* 0.69 | *p_interaction_ =* 0.5967 |
| Change | *p_interaction_ =* 0.0233 | *p_interaction_ =* 0.2296 | *p_interaction_ =* 0.2532 | *p_interaction_ =* 0.2532 | *p_interaction_ =* 0.1251 | *p_interaction_ =* 0.9209 |
| Nitrogen dioxide |  |  |  |  |  |  |
| Mean | *p_interaction_ =* 0.7653 | *p_interaction_ =* 0.4594 | *p_interaction_ =* 0.4113 | *p_interaction_ =* 0.4113 | *p_interaction_ =* 0.1321 | *p_interaction_ =* 0.5924 |
| Maximum | *p_interaction_ =* 0.999 | *p_interaction_ =* 0.8867 | *p_interaction_ =* 0.4976 | *p_interaction_ =* 0.4976 | *p_interaction_ =* 0.4594 | *p_interaction_ =* 0.7407 |
| Minimum | *p_interaction_ =* 0.5611 | *p_interaction_ =* 0.4535 | *p_interaction_ =* 0.3097 | *p_interaction_ =* 0.3097 | *p_interaction_ =* 0.0967 | *p_interaction_ =* 0.3724 |
| Change | *p_interaction_ =* 0.8495 | *p_interaction_ =* 0.5918 | *p_interaction_ =* 0.6743 | *p_interaction_ =* 0.6743 | *p_interaction_ =* 0.8312 | *p_interaction_ =* 0.5728 |
| Ozone |  |  |  |  |  |  |
| Mean | *p_interaction_ =* 0.995 | *p_interaction_ =* 0.2133 | *p_interaction_ =* 0.9693 | *p_interaction_ =* 0.9693 | *p_interaction_ =* 0.9411 | *p_interaction_ =* 0.9662 |
| Maximum | *p_interaction_ =* 0.8849 | *p_interaction_ =* 0.2666 | *p_interaction_ =* 0.7396 | *p_interaction_ =* 0.7396 | *p_interaction_ =* 0.793 | *p_interaction_ =* 0.5433 |
| Minimum | *p_interaction_ =* 0.8842 | *p_interaction_ =* 0.8459 | *p_interaction_ =* 0.9244 | *p_interaction_ =* 0.9244 | *p_interaction_ =* 0.9325 | *p_interaction_ =* 0.8296 |
| Change | *p_interaction_ =* 0.9688 | *p_interaction_ =* 0.2747 | *p_interaction_ =* 0.6007 | *p_interaction_ =* 0.6007 | *p_interaction_ =* 0.6192 | *p_interaction_ =* 0.504 |
| Carbon monoxide |  |  |  |  |  |  |
| Mean | *p_interaction_ =* 0.7027 | *p_interaction_ =* 0.7526 | *p_interaction_ =* 0.7512 | *p_interaction_ =* 0.7512 | *p_interaction_ =* 0.3037 | *p_interaction_ =* 0.647 |
| Maximum | *p_interaction_ =* 0.9758 | *p_interaction_ =* 0.7414 | *p_interaction_ =* 0.8174 | *p_interaction_ =* 0.8174 | *p_interaction_ =* 0.461 | *p_interaction_ =* 0.892 |
| Minimum | *p_interaction_ =* 0.2778 | *p_interaction_ =* 0.8218 | *p_interaction_ =* 0.5548 | *p_interaction_ =* 0.5548 | *p_interaction_ =* 0.1836 | *p_interaction_ =* 0.5776 |
| Change | *p_interaction_ =* 0.7617 | *p_interaction_ =* 0.7837 | *p_interaction_ =* 0.9626 | *p_interaction_ =* 0.9626 | *p_interaction_ =* 0.6797 | *p_interaction_ =* 0.9636 |
| Temperature |  |  |  |  |  |  |
| Mean | *p_interaction_ =* 0.5558 | *p_interaction_ =* 0.606 | *p_interaction_ =* 0.7026 | *p_interaction_ =* 0.7026 | *p_interaction_ =* 0.9246 | *p_interaction_ =* 0.9374 |
| Maximum | *p_interaction_ =* 0.5057 | *p_interaction_ =* 0.4388 | *p_interaction_ =* 0.7577 | *p_interaction_ =* 0.7577 | *p_interaction_ =* 0.9459 | *p_interaction_ =* 0.8196 |
| Minimum | *p_interaction_ =* 0.5123 | *p_interaction_ =* 0.7312 | *p_interaction_ =* 0.8406 | *p_interaction_ =* 0.8406 | *p_interaction_ =* 0.926 | *p_interaction_ =* 0.7327 |
| Change | *p_interaction_ =* 0.9552 | *p_interaction_ =* 0.0457 | *p_interaction_ =* 0.5685 | *p_interaction_ =* 0.5685 | *p_interaction_ =* 0.3841 | *p_interaction_ =* 0.0071 |
| Dew point temperature |  |  |  |  |  |  |
| Mean | *p*_interaction_ = 0.7479 | *p_interaction_ =* 0.9443 | *p_interaction_ =* 0.8669 | *p_interaction_ =* 0.8669 | *p_interaction_ =* 0.5409 | *p_interaction_ =* 0.5541 |
| Maximum | *p_interaction_ =* 0.7887 | *p_interaction_ =* 0.9479 | *p_interaction_ =* 0.9184 | *p_interaction_ =* 0.9184 | *p_interaction_ =* 0.5386 | *p_interaction_ =* 0.7415 |
| Minimum | *p_interaction_ =* 0.7002 | *p_interaction_ =* 0.9309 | *p_interaction_ =* 0.9031 | *p_interaction_ =* 0.9031 | *p_interaction_ =* 0.5391 | *p_interaction_ =* 0.4481 |
| Change | *p_interaction_ =* 0.5621 | *p_interaction_ =* 0.4623 | *p_interaction_ =* 0.8573 | *p_interaction_ =* 0.8573 | *p_interaction_ =* 0.8128 | *p_interaction_ =* 0.0319 |
| Atmospheric pressure |  |  |  |  |  |  |
| Mean | *p_interaction_ =* 0.0506 | *p_interaction_ =* 0.2763 | *p_interaction_ =* 0.1928 | *p_interaction_ =* 0.1928 | *p_interaction_ =* 0.0007 | *p_interaction_ =* 0.109 |
| Maximum | *p_interaction_ =* 0.4018 | *p_interaction_ =* 0.4543 | *p_interaction_ =* 0.8432 | *p_interaction_ =* 0.8432 | *p_interaction_ =* 0.5265 | *p_interaction_ =* 0.7485 |
| Minimum | *p_interaction_ =* 0.0286 | *p_interaction_ =* 0.3214 | *p_interaction_ =* 0.087 | *p_interaction_ =* 0.087 | *p_interaction_ =* 0.0003 | *p_interaction_ =* 0.5238 |
| Change | *p_interaction_ =* 0.4741 | *p_interaction_ =* 0.735 | *p_interaction_ =* 0.676 | *p_interaction_ =* 0.676 | *p_interaction_ =* 0.667 | *p_interaction_ =* 0.3331 |
| Cloud amount | *p_interaction_ =* 0.4207 | *p_interaction_ =* 0.1822 | *p_interaction_ =* 0.0382 | *p_interaction_ =* 0.0382 | *p_interaction_ =* 0.12 | *p_interaction_ =* 0.1838 |
